# *Orientia tsutsugamushi* Subverts Dendritic Cell Functions by Escaping from Autophagy and Impairing Their Migration

**DOI:** 10.1371/journal.pntd.0001981

**Published:** 2013-01-03

**Authors:** Ji-Hye Choi, Taek-Chin Cheong, Na-Young Ha, Youngho Ko, Chung-Hyun Cho, Ju-Hong Jeon, Insuk So, In-Kyu Kim, Myung-Sik Choi, Ik-Sang Kim, Nam-Hyuk Cho

**Affiliations:** 1 Department of Microbiology and Immunology, Seoul National University College of Medicine, Seoul, Republic of Korea; 2 Pharmacology, Seoul National University College of Medicine, Seoul, Republic of Korea; 3 Physiology, Seoul National University College of Medicine, Seoul, Republic of Korea; 4 Biochemistry and Molecular Biology, Seoul National University College of Medicine, Seoul, Republic of Korea; 5 Institute of Endemic Disease, Seoul National University Medical Research Center and Bundang Hospital, Jongno-Gu, Seoul, Republic of Korea; National Institutes of Health, United States of America

## Abstract

**Background:**

Dendritic cells (DCs) are the most potent antigen-presenting cells that link innate and adaptive immune responses, playing a pivotal role in triggering antigen-specific immunity. Antigen uptake by DCs induces maturational changes that include increased surface expression of major histocompatibility complex (MHC) and costimulatory molecules. In addition, DCs actively migrate to regional lymph nodes and activate antigen-specific naive T cells after capturing antigens. We characterize the functional changes of DCs infected with *Orientia tsutsugamushi*, the causative agent of scrub typhus, since there is limited knowledge of the role played by DCs in *O. tsutsugamushi* infection.

**Methodology/Principal Finding:**

*O. tsutsugamushi* efficiently infected bone marrow-derived DCs and induced surface expression of MHC II and costimulatory molecules. In addition, *O. tsutsugamushi* induced autophagy activation, but actively escaped from this innate defense system. Infected DCs also secreted cytokines and chemokines such as IL-6, IL-12, MCP5, MIP-1α, and RANTES. Furthermore, *in vitro* migration of DCs in the presence of a CCL19 gradient within a 3D collagen matrix was drastically impaired when infected with *O. tsutsugamushi*. The infected cells migrated much less efficiently into lymphatic vessels of ear dermis *ex vivo* when compared to LPS-stimulated DCs. *In vivo* migration of *O. tsutsugamushi*-infected DCs to regional lymph nodes was significantly impaired and similar to that of immature DCs. Finally, we found that MAP kinases involved in chemotactic signaling were differentially activated in *O. tsutsugamushi*-infected DCs.

**Conclusion/Significance:**

These results suggest that *O. tsutsugamushi* can target DCs to exploit these sentinel cells as replication reservoirs and delay or impair the functional maturation of DCs during the bacterial infection in mammals.

## Author Summary

Scrub typhus is an acute febrile illness caused by *Orientia tsutsugamushi* infection and is one of the main causes of febrile illness in the Asia-Pacific region. If not properly treated with antibiotics, patients often develop severe vasculitis that affects multiple organs, and the mortality rate of untreated patients reaches up to 30%. To understand the pathogenic mechanisms of the infectious disease, we characterized the functional changes of *O. tsutsugamushi*–infected dendritic cells (DCs), which play a pivotal role in orchestrating innate and adaptive immune responses. The obligate intracellular bacteria efficiently infected bone marrow-derived DCs and activated the cells as measured by induced surface expression of MHC II and costimulatory molecules, secretion of cytokines and chemokines, and autophagy induction. However, the live bacteria actively escaped from host autophagosomes and the migration of infected cells was severely impaired *in vitro*, *ex vivo*, and *in vivo* infection models. Finally, we found that MAP kinases involved in chemotactic signaling were differentially activated in *O. tsutsugamushi*-infected DCs. These results suggest that *O. tsutsugamushi* can target DCs to exploit these sentinel cells as replication reservoirs and delay or impair the functional maturation of DCs during the bacterial infection in mammals.

## Introduction

Dendritic cells (DCs) are the most potent antigen-presenting cells (APCs) that initiate and orchestrate immune responses [1]. Upon pathogen infection, DCs capture foreign antigen and undergo maturational changes including increased surface expression of major histocompatibility complex (MHC) and costimulatory molecules, such as CD40, CD80, and CD86. Moreover, they migrate from peripheral tissues via afferent lymphatic vessels into draining lymph nodes where they prime antigen-specific naive T cells [2]. Migration of DCs to regional lymph nodes is mainly regulated by changes in surface expression of chemokine receptors. Increased surface expression of CCR7 during DC maturation enables DCs to respond to the lymphoid chemokines, CCL19 and CCL21, which are constitutively produced by lymphatic endothelial cells and secondary lymphoid organs. Therefore, surface expression of CCR7, in addition to the expression of MHC and costimulatory molecules, is critical for initiating antigen-specific T cell responses in regional lymph nodes.

Infectious microbial pathogens have established numerous strategies that disrupt and confound DC functions to survive and evade host immune antimicrobial mechanisms [3]. For example, secondary lymphoid organs of human immunodeficiency virus (HIV)-infected individuals have been shown to contain an accumulation of semi-mature dendritic cells that exhibit a lower expression of costimulatory molecules that support differentiation of CD4^+^ T cells into regulatory T cells and suppress effector functions [4]. Herpes simplex virus type 1 infection rapidly degrades cytohesin-interacting protein in DCs and impairs DC migration through increased integrin-mediated adhesion [5]. DCs infected with human respiratory syncytial virus do not efficiently increase CCR7 expression and hence displayed inefficient chemotatic migration toward a CCL19 gradient [6]. Filamentous hamagglutinin of *Bordetella pertusis* inhibits IL-12 and stimulates IL-10 production by DCs, which directs naive T cells to differentiate into regulatory subtypes [7]. These diverse hijacking strategies employed by microbial pathogens to utilize DCs for their own benefit may have been acquired during their eternal struggle for evolutionary survival.

*Orientia tsutsugamushi*, the causative agent of scrub typhus, is an obligate intracellular bacterium [8]. The bacteria are transmitted from chigger mites to humans, after which *O. tsutsugamushi* invades cells in the dermis, causing an inflammatory lesion called an eschar [9]. A recent study using eschar skin biopsies from scrub typhus patients showed that *O. tsutsugamushi* has tropism for DCs and monocytes rather than endothelial cells, traditionally regarded to be the primary target of the bacterial pathogen [9]. Immunohistological analysis of eschar lesions revealed that DCs and macrophages predominantly infiltrate at the dermo-epidermal junction while the bacterial pathogen is mainly within Langerhan's cells, dermal DCs, and activated macrophages [9]. These results suggest that infection of dendritic cells and macrophages may be a potential route for dissemination of *O. tsutsugamushi* from the initial infection site and that cellular tropism may influence its interaction with host immune responses.

Currently, there is limited knowledge of the role played by DCs in *O. tsutsugamushi* infection. Therefore, we investigated DC responses to *O. tsutsugamushi* infection for the first time. Although *O. tsutsugamushi* is capable of inducing humoral and cellular immune responses *in vivo*, it is not clear whether *O. tsutsugamushi*-infected DCs are successfully activated and migrate to secondary lymphoid organs to initiate anti-bacterial immune responses. This study was designed to evaluate the functional interaction of *O. tsutsugamushi* with DCs to better our understanding of the immunological pathogenesis of *O. tsutsugamushi* during the early phase of infection.

## Materials and Methods

### Ethics Statement

Animal experiments were approved by the Seoul National University Institutional Animal Care and Use Committee (SNU IACUC, Permit No. SNU-100414-1) and performed in strict accordance with the recommendations in the National Guide Line for the care and use of laboratory animals.

### Mice

C57BL/10NAGCSnAi-(KO) Rag2 (H-2b) mice (Taconic Farms, Germantown, NY) and C57BL/6 mice (Orient Bio, Seongnam, South Korea) were housed and maintained in the specific pathogen-free facility at Seoul National University (SNU) College of Medicine.

### Preparation of *O. tsutsugamushi*

The Boryong strain [8], [10], [11] of *O. tsutsugamushi* was purified using a modified Percoll gradient purification method [12]. *O. tsutsugamushi* was propagated in L929 cells. At 3 to 4 days postinfection, infectivity was determined using an indirect immunofluorescence assay. When an infection rate of >90% was achieved, the cells were harvested by centrifugation at 6,000× *g* for 20 min. The cell pellet was resuspended with 6.5 ml of Tris-sucrose (TS) buffer (33 mM Tris-Cl [pH 7.4], 0.25 M sucrose) and the cells were homogenized using 100 strokes of a Polytron homogenizer (Wheaton Inc., Millville, NJ) followed by centrifugation at 200× *g* for 5 min. The supernatant was then mixed with 40% Percoll (Pharmacia Fine Chemicals, Uppsala, Sweden) in TS buffer and centrifuged at 25,000× *g* for 60 min. The bacterial band was collected and centrifuged at 77,000× *g* for 30 min. The bacterial pellet was washed 3 times in TS buffer, resuspended in culture media, and stored in liquid nitrogen until use. The infectivity titer of the inoculum was determined as previously described [13], [14]. For infection assays, 2.5×10^6^ infected-cell counting unit (ICU) [14] of *O. tsutsugamushi* was to infect cell cultures in 24 well plate (∼10 bacteria/cell). The same amount of *O. tsutsugamushi* were also inactivated by exposing the bacteria to a 30-W UV lamp for 30 min in 6-well plates [15] or by heating the bacterial stocks at 100°C for 10 min [13], and used as control infection. Unstimulated immature DCs were also used as negative control in each experiment.

### Generation of Bone-Marrow-Derived DCs

DCs were generated from the bone marrow of 6- to 12-week-old Rag2 knock-out mice. The bone marrow cells were flushed out of the femurs and tibias with serum-free Iscove's modified Eagle medium (IMDM; Gibco Invitrogen, Grand Island, NY). The single cell suspension was then filtered through a nylon cell strainer (70-µm Nylon mesh; BD Biosciences), washed twice with complete IMDM [supplemented with 10% FBS, recombinant mouse GM-CSF (1.5 ng/ml; PeproTech, Rocky Hill, NJ) and mouse IL-4 (1.5 ng/ml; PeproTech), penicillin (100 units/ml), streptomycin (100 µg/ml), gentamicin (50 µg/ml), L-glutamine (2 mM), and β-mercaptoethanol (50 nM; Gibco Invitrogen)], and seeded at a concentration of 1×10^6^ cells per well in a 24-well plate. Half of the medium was replaced every other day with an equal volume of complete IMDM medium for 6 days [16]. The immature DCs generated were stimulated with *O. tsutsugamushi* or 0.5 µg/ml Lipopolysaccharide (LPS; Sigma Aldrich, St. Louis, MO) for 20 h. In every infection study, we confirmed that more than 90% of the cells were infected with *O. tsutsugamushi* after 20 h of incubation.

### Immunofluorescence Microscopy

Immunofluorescence microscopy was used to visualize *O. tsutsugamushi* [12]. Briefly, infected cells were fixed in PBS containing 4% paraformaldehyde for 15 min at room temperature and permeabilized in 0.2% Triton X-100 for 15 min. Cells infected with *O. tsutsugamushi* were incubated with pooled sera from scrub typhus patients for 1 h, followed by incubation with AlexaFluor488-conjugated goat anti-human IgG (Molecular Probes). For autophagy detection, cells were co-stained with anti-LC3 antibody (NB100-2220; Novus Biologicals, Littleton, CO) and AlexaFluor594-conjugated secondary antibody (Molecular Probes). Immunostained cells were examined under an Olympus FV1000 laser scanning confocal microscope (Olympus; Tokyo, Japan). All images were analyzed and processed using the Olympus Fluoview software (Olympus).

### Flow Cytometric Analysis

In order to investigate the responses of DCs after bacterial infection, immature DCs were incubated with live or inactivated *O. tsutsugamushi* (∼10 bacteria/cell), or *E. coli* LPS (0.5 µg/ml) as a positive control. In some experiments, cells were infected with different doses (10 bacteria/cell or 20 bacteria/cell) or stimulated with inactivated bacteria as mentioned above. At 20 h after stimulation, DCs were stained with antibodies against the indicated surface molecules or isotype control antibodies after blocking Fc receptors with anti-CD16/32 (2.4G2; BD Pharmingen). Allophycocyanin (APC) Cy7-conjugated anti-CD11c (N418; Biolegend, San Diego, CA), isothiocyanate (FITC)-conjugated anti-I-A[b] (AF6-120.1; BD Pharmingen, Franklin Lakes, NJ), phycoerythrin (PE)-conjugated anti-CD40 (3/23; BD Pharmingen), APC-conjugated anti-CD80 (1610A1; eBioscience, San Diego, CA), PE Cy7-conjugated anti-CD86 (GL-1; eBioscience), PE-conjugated anti-CCR7 (4B12; eBioscience), and 7-AAD (BD Pharmingen) were used for flow cytometric analysis. Fluorescence intensities of the stained molecules were examined after gating 7-AAD-negative and CD11c-positive live DCs on a FACSCanto II flow cytometer (BD Biosciences). Data were analyzed using Flowjo software (Tree Star, Ashland, OR).

### Cytokine Antibody Array

DCs were infected with *O. tsutsugamushi* for 20 h and the culture supernatant was used for cytokine antibody array. The culture supernatant from unstimulated immature DCs was used as negative control. We used RayBio Mouse Cytokine Antibody Array III & 3.1 (RayBiotech, Inc., Norcross, GA), which can simultaneously detects 62 proteins (Axl, BLC, CD30 L, CD30, CD40, CRG-2, CTACK, CXCL16, Eotaxin, Eotaxin-2, Fas Ligand, Fractalkine, GCSF, GM-CSF, IFN-γ, IGFBP-3, IGFBP-5, IGFBP-6, IL-1β, IL-10, IL-12 p40/p70, IL-12 p70, IL-13, IL-17, IL-1α, IL-2, IL-3, IL-3 Rβ, IL-4, IL-5, IL-6, IL-9, KC/CXCL1, Leptin/OB, Leptin R, LIX, L-Selectin, Lymphotactin, MCP1, MCP-5, M-CSF, MIG, MIP-1α, MIP-1γ, MIP-2, MIP-3β, MIP-3α, PF-4, P-Selectin, RANTES, SCF, SDF-1α, sTNF RI, sTNF RII, TARC, TCA-3, TECK, TIMP-1, TNF-α, Thrombopoietin, VCAM-1, and VEGF-A), as recommended by the manufacturer's instructions. Briefly, each membrane was blocked in 2 ml of Blocking Buffer and incubated at room temperature (RT) for 30 min. Membranes were then incubated with 1 ml of culture supernatants at RT for 2 h, washed three times with 2 ml of Wash Buffer I at RT, and further washed twice with 2 ml of 1× Wash Buffer II at RT. 1∶250-diluted biotin-conjugated primary antibodies were added. The membranes were incubated at 4°C overnight, washed as described above, incubated with 1∶1000-diluted HRP-conjugated streptavidin at RT for 1 h, and washed with wash buffer three times. After incubation with Detection solution, the membrane images were analyzed by LAS-3000 (Fujifilm, Tokyo, Japan). Signal intensities were analyzed by Quantity One software (Bio-Rad, Hercules, CA).

### 3D Collagen Gel Chemotaxis Assay

DCs unstimulated or stimulated with indicated agents were mixed with PureCol (Advanced biomatrix, Poway, CA) in 10× PBS, resulting in gels with a collagen concentration of 1.5 mg/ml. Final concentration of the cells in the assay was 1×10^7^ cells/ml. The collagen-cell mixture was cast in 15 μ-slide VI flat (ibidi, München, Germany) and incubated at 37°C for 40 min. After assembly of the collagen fibers, recombinant chemokine CCL19 (1.2 µg/ml) (R&D Systems, Inc., Minneapolis, MN) diluted in IMDM containing 10% FBS and PureCol mixture were cast on the opposite side of the slide. For cell tracking, cells were visualized by time lapse imaging using a confocal microscope (Olympus). Manual single cell tracking of samples was performed using Manual Tracking Plugin of Image J (National Institute of Mental Health, Bethesda, MD). Cells were tracked every 2 min per frame for 4 h. Velocity, Euclidean distance and directionality parameters were calculated and visualized as plots [17].

### Ear Crawl-In Assay

Ears were obtained from sacrificed C57BL/6 mice. The ears were mechanically split into dorsal and ventral halves and mounted on 24 well plates with the dermal surface exposed [18]. Unstimulated immature DCs or DCs (10^6^ cell/ml) stimulated with *O. tsutsugamushi* or LPS for 20 h were labeled with 5-(and 6)-carboxyfluorescein diacetate succinimidyl ester (CFSE; Invitrogen). Cells were washed twice with PBS, incubated with CFSE (5 µM) for 10 min at 37°C, and washed twice with PBS containing 0.5% of BSA. DCs were resuspended in culture medium, added on top of the dermis, and incubated at 37°C for 2 h. After gently washing away non-infiltrated DCs, the ears were fixed with 4% paraformaldehyde (Sigma) for 30 min. The ears were then incubated with a rat monoclonal anti-LYVE-1 antibody (R&D Systems) at 4°C overnight, washed with PBS and stained with Alexa fluor 647-conjugated anti-rat secondary antibody (Molecular Probes).

### *In vivo* Migration Assay

C57BL/6 mice were preinjected with LPS (0.3 µg/leg) in the hind-leg footpad one day before injection of DCs [19]. DCs (10^6^ cell/ml) were stimulated with *O. tsutsugamushi* or LPS (0.5 µg/ml) for 20 h, labeled with CFSE, and resuspended in PBS to a concentration of 10^8^ cells/ml. Unstimulated immature DCs were also included as negative control. 30 µl of the cell solution was injected into the footpad of C57/BL6 mice. 48 h later, popliteal lymph nodes were collected and treated with 1 mg/ml of collagenase D (Sigma) at 37°C for 40 min to collect the cells from the lymph nodes. The percentage of migrated DCs in total lymph node cells was determined by FACS analysis [20].

### Immunoblot Analysis

DCs stimulated with CCL19 (200 ng/mL) for the indicated time periods were washed twice with ice-cold PBS. Cells were lysed with lysis buffer (50 mM Tris-HCl [pH 7.5], 150 mM NaCl, 1% Triton X-100, 1% sodium deoxycholate, 0.1% sodium dodecyl sulphate (SDS), and 2 mM EDTA). The cellular proteins separated by SDS–polyacrylamide gel electrophoresis were electrotransferred to PVDF membranes and subjected to immunoblot analysis using the indicated antibodies. anti-LC3 (NB100-2220; Novus Biologicals, Littleton, CO), anti-p44/42 MAPK (ERK1/2) (3A7; Cell Signaling Technology, Frankfurt, Germany), anti-phospho-ERK (E-4; Santa Cruz), anti-p38α/β (A-12; Santa Cruz), anti-phospho-p38 (D-8; Santa Cruz), anti-GAPDH (6C5; Santa Cruz), Horseradish peroxidase–conjugated anti-mouse antibody (Santa Cruz Biotechnology, Inc., Santa Cruz, CA), were used for immunoblot analysis. The immune-reactive bands were detected using enhanced chemiluminescence reagents (Ab frontier, Seoul, South Korea). Signal intensities were analyzed by Quantity One software (Bio-Rad, Hercules, CA).

### Statistical Analysis

Statistical analysis of all the experimental data was performed using the two-tailed Student's *t*-test with 95% confidence interval. Data are expressed as the mean ± standard deviation and a *p* value<0.05 was considered to be statistically significant. Statistical analyses were accomplished using GraphPad Prism 5.01 (GraphPad Software Inc., La Jolla, CA).

## Results

### Activation of DCs Infected with *O. tsutsugamushi*

**Figure 1:**
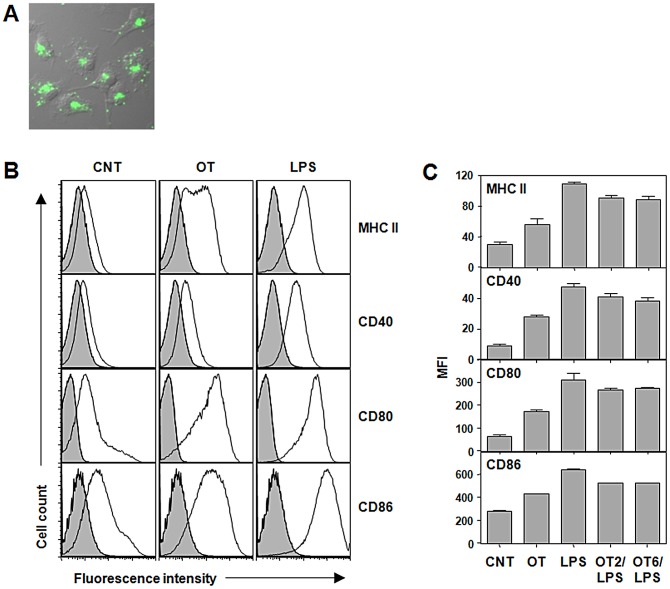
Activation of DCs infected with *O. tsutsugamushi in vitro*. A. DCs were infected with *O. tsutsugamushi* for 24 h and stained with pooled scrub typhus patients' sera (green). The immunofluorescence image was merged with DIC image of the cells. B. DCs were stimulated with *O. tsutsugamushi* or LPS (0.5 µg/ml) for 20 h, stained with antibodies against the indicated surface molecules, and then analyzed by flow cytometer. Representative histograms of CD11c^+^-gated cells are presented. Gray filled: isotype control. C. The mean fluorescent intensity (MFI) of the surface markers from three separate experiments are presented. Error bar: mean+S.D., CNT: immature DCs, OT: DCs infected with *O. tsutsugamushi*, LPS: DCs stimulated LPS, OT2/LPS or OT6/LPS: DCs stimulated *O. tsutsugamushi* for 2 h (OT2) or 6 h (OT6) and then stimulated with LPS.

**Figure 2:**
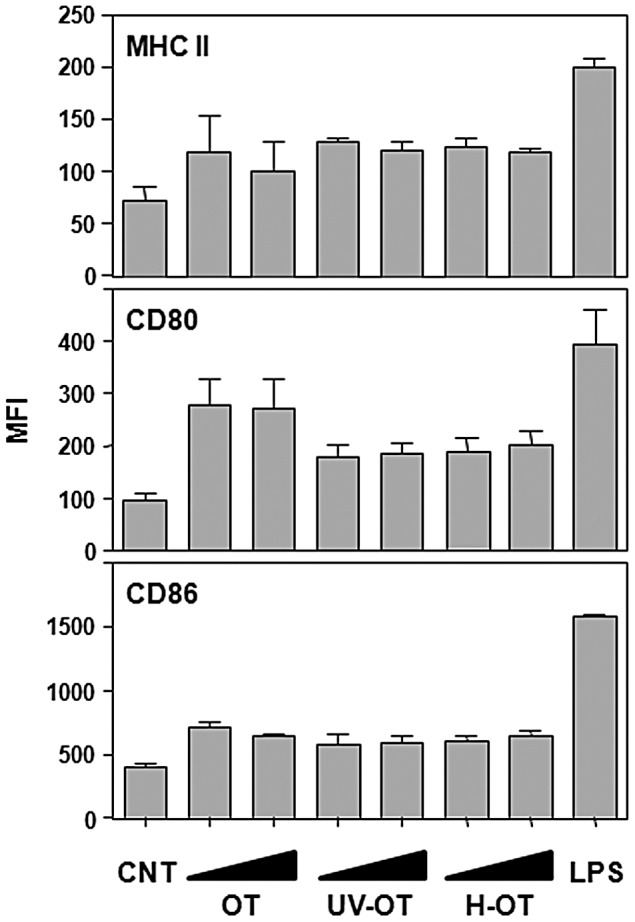
Activation of DCs with increasing doses of live or inactivated *O. tsutsugamushi*. DCs were stimulated with different doses (10 or 20 bacteria/cell) of live *O. tsutsugamushi* (OT), UV-inactive *O. tsutsugamushi* (UV-OT), heat-killed *O. tsutsugamushi* (H-OT), or LPS (0.5 µg/ml) for 20 h, stained with antibodies against the indicated surface markers, and analyzed by flow cytometric analysis. The mean fluorescent intensities (MFI) of the surface markers from three separate experiments are presented. Error bar: mean+S.D., CNT: immature DCs.

Since *O. tsutsugamushi* has been detected in DCs in eschars of human scrub typhus patients [9], we first confirmed whether the bacteria are able to infect and replicate within DCs. Bone marrow-derived immature DCs were infected with *O. tsutsugamushi* and further incubated for one day. Efficient replication of *O. tsutsugamushi* in the perinuclear region [21] was observed using indirect immunofluorescence assay (Figure 1A). In order to investigate the responses of DCs after bacterial infection, immature DCs were incubated with *O. tsutsugamushi* or *E. coli* LPS as a positive control. Surface expression of MHC II, CD40, CD80, and CD86 were significantly (*p*<0.05) up-regulated at 20 h after infection with *O. tsutsugamushi*, even though the levels of expression were lower than those of cells stimulated with LPS (Figure 1B and 1C). Mean fluorescence values of MHC II, CD40, and CD80 in *O. tsutsugamushi*-infected DCs increased by more than 2 fold when compared to those of un-stimulated immature DCs, indicating that bacterial infection induced DC activation. Since the levels of surface expression of activation markers were lower than those of cells stimulated with LPS, we further examined whether this is dose-dependent by increasing the amount of bacterial inoculums. In addition, we also evaluated whether active bacterial replication was required for DC activation by exposing DCs to the same amount of heat or UV-inactivated bacteria. As shown in Figure 2, surface expression of MHC II and CD86 did not significantly change with increased of live or inactivated bacteria, whereas CD80 expression in DCs incubated with inactivated bacteria was slightly reduced when compared to that of live *O. tsutsugamushi*.

Possible explanations for the weaker induction of costimulatory molecules by *O. tsutsugamushi* infection compared to LPS stimulation could be a direct inhibition or insufficient stimulation by the intracellular pathogen. We therefore investigated whether the exposure of *O. tsutsugamushi*-infected DCs to a secondary stimulation with LPS would result in a more efficient induction of costimulatory molecules. DCs were infected with *O. tsutsugamushi* for 2 or 6 h, followed by a secondary stimulation with LPS. The surface expression levels of costimulatory molecules and MHC II were quantified at 20 h after infection (Figure 1C, OT2/LPS and OT6/LPS). The secondary treatment with LPS induced a significant increase of costimulatory molecules on the surface of infected DCs, thus demonstrating that the relatively low levels of surface expression of costimulatory molecules on *O. tsutsugamushi*-infected DCs is due to an insufficient stimulation of DCs rather than a direct inhibition by the intracellular pathogen.

**Figure 3:**
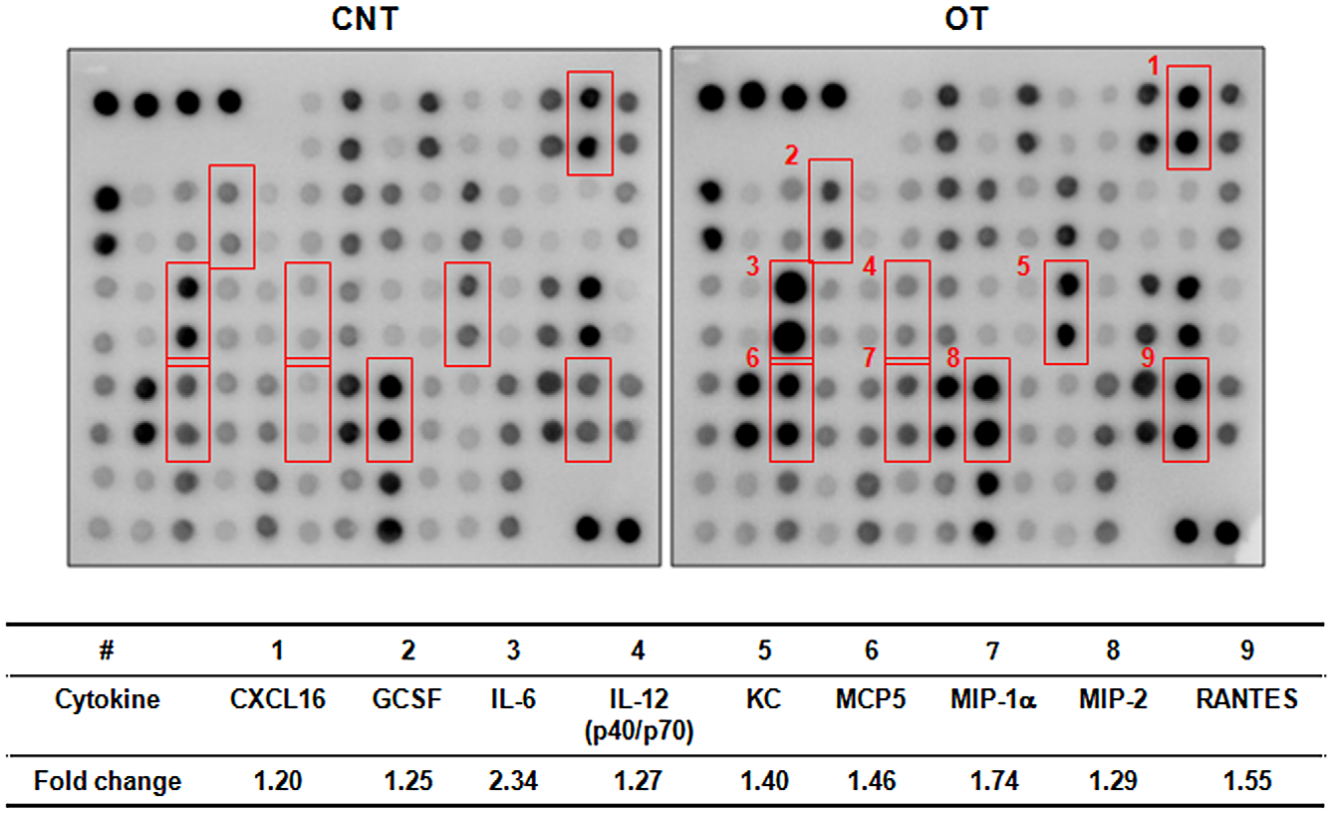
Cytokine profiling of DCs infected with *O. tsutsugamushi*. Secreted cytokines and chemokines from infected DCs were analyzed using a cytokine antibody array that simultaneously detects 62 cytokines and chemokines. Unstimulated immature DCs were used as a negative control (CNT). The membrane images were analyzed for the changes in signal intensities (top). Cytokines/chemokines with a normalized fold change greater than 1.2 are summarized (bottom). CNT: immature DC, OT: DCs infected with *O. tsutsugamushi*.

*O. tsutsugamushi* also stimulates the production of proinflammatory cytokines and chemokines that are important in both innate and acquired immunity. In order to examine the inflammatory mediators released by *O. tsutsugamushi*-infected DCs, we compared the expression profiles of cytokines and chemokines of unstimulated and *O. tsutsugamushi*-infected DCs using a cytokine antibody array. We observed a more than 20% up-regulation of nine cytokines and chemokines in DCs infected with *O. tsutsugamushi* compared to unstimulated immature DCs (Figure 3). Among them, IL-6, MIP-1α, and RANTES increased by more than 50% in *O. tsutsugamushi*-infected cells. These results further demonstrate that *O. tsutsugamushi* infection activates DCs.

### Active Induction but Escape of *O. tsutsugamushi* from Autophagy

**Figure 4:**
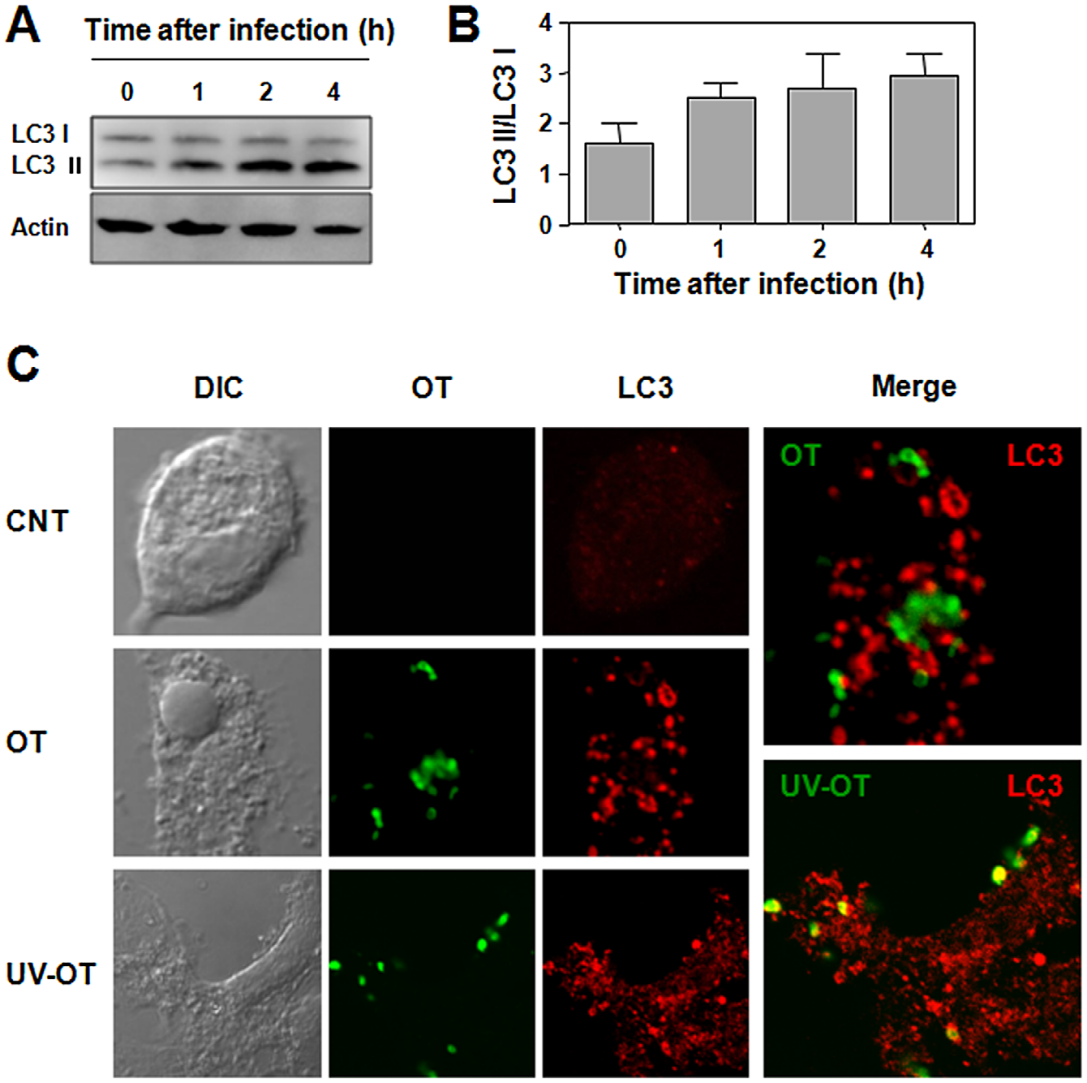
Induction of autophagy in DCs infected with *O. tsutsugamushi*. A and B. DCs were infected with *O. tsutsugamushi* for indicated time periods and subjected to immunoblot analysis for LC3 and β-actin as a loading control. LC3 II/LC3 I ratios were determined by densitometry of the immunoblot results from three independent experiments. Error bar: mean+S.D. C. DCs were infected with live *O. tsutsugamushi* (OT) or UV-inactivated bacteria (UV-OT) for 2 h and stained with scrub typhus patients' sera and anti-LC3 antibody. Colocalization of *O. tsutsugamushi* (green) with autophagosomes (red) was analyzed by confocal microscopy. CNT: immature DC, DIC: differential interference contrast.

Autophagy is an innate defense mechanism against various intracellular pathogens. To survive within host cells, intracellular pathogens have evolved mechanisms to avoid elimination by autophagy [22]. To analyze whether infection with *O. tsutsugamushi* can stimulate autophagy in DCs, we infected cells with the bacteria and measured the conversion of LC3-I to LC3-II up to 4 h after infection (Figure 4A and B). The conversion of LC3-1 to LC3-II gradually increased when DCs were infected with *O. tsutsugamushi*. Induction of autophagy was also monitored by confocal laser-scanning microscopy analysis in DCs. Infection of *O. tsutsugamushi* led to a drastic increase of intracellular autophagosomes (Figure 4C). Interestingly, most of the intracellular bacteria did not co-localized with LC3-positive autophagosomes throughout the infection, indicating that few bacteria are actually captured. To further investigate whether *O. tsutsugamushi* actively evades autophagy, we treated DCs with UV-inactivated bacteria. Even though UV-treated bacteria induced autophagosome formation within DCs as efficiently as live bacteria, all the inactivated bacterial particles co-localized with LC3-positive autophagosomes in contrast to live bacteria. These results indicate that live *O. tsutsugamushi* actively evades the cellular autophagic system although the bacteria activate cellular autophagy upon infection regardless of its viability.

### Impaired Migration of DCs Infected with *O. tsutsugamushi*

**Figure 5:**
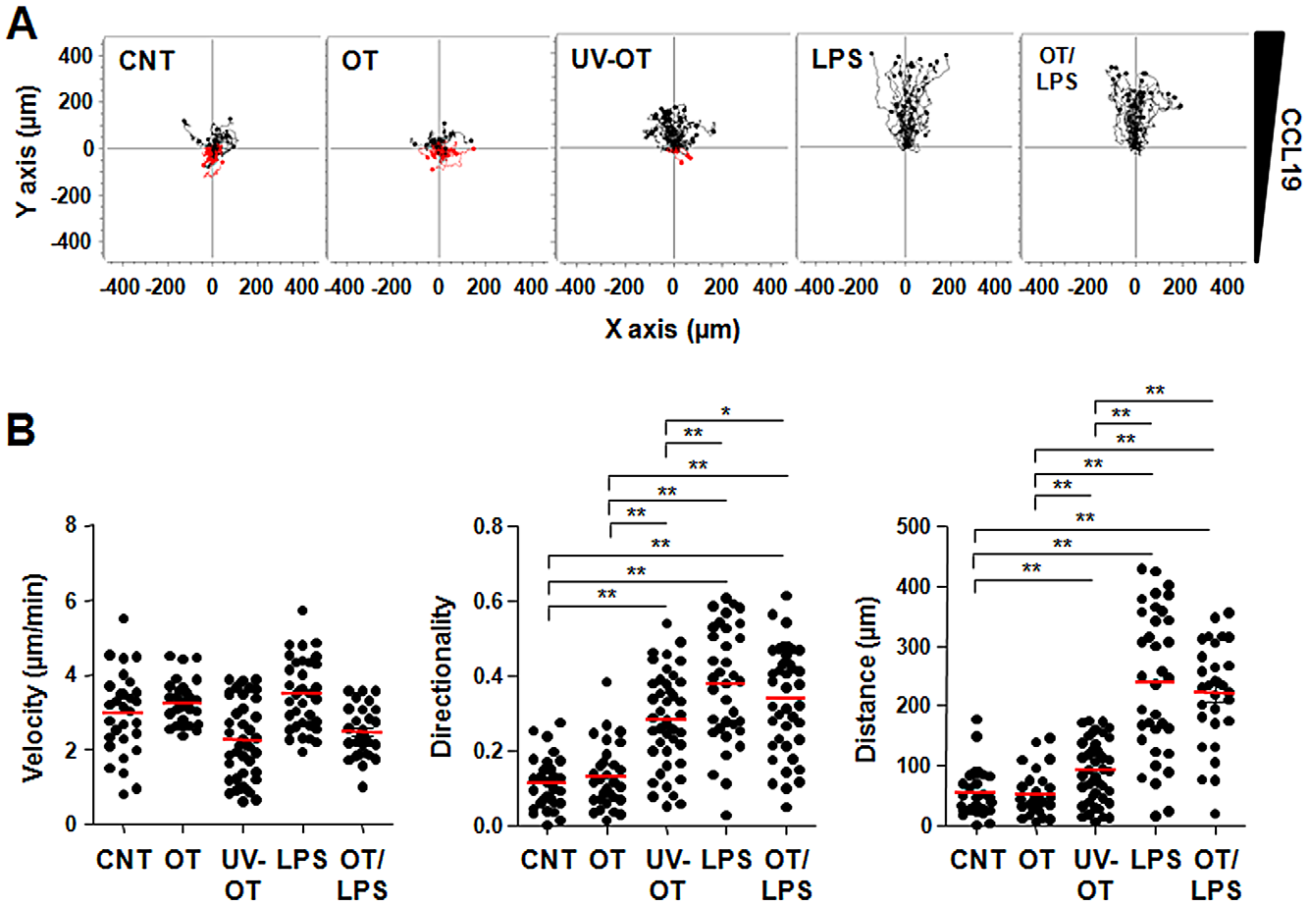
*In vitro* migration of DCs infected with *O. tsutsugamushi* in a 3D collagen matrix. A. Single cell tracking was performed using Manual Tracking Plugin with Image J software. Thirty cells were randomly selected and tracked for 4 h. B. Speed, directionality, and Euclidean distance parameters were calculated by analyzing the acquired data from the Chemotaxis and Migration Tool Plugin software. The graphs represent velocity, directionality, and Euclidean distance, respectively. Red bars represent mean values. *: *p*<0.05, **: *p*<0.01, CNT: immature DCs, OT: DCs infected with *O. tsutsugamushi*, UV-OT: DCs infected with UV-inactivated *O. tsutsugamushi*, LPS: DCs stimulated with LPS, OT/LPS: DCs stimulated with *O. tsutsugamushi* and LPS.

The ability of activated DCs to migrate to secondary lymphoid organs where naive T cells reside is a crucial step in the generation of primary T cell responses. DC migration to regional lymph nodes is a complex process composed of multiple steps, including movement to the tissue interstitium, entry into lymphatic vessels, and extravasation from the lymphatic system into the lymph nodes [2]. In order to investigate the effect of *O. tsutsugamushi* infection on DC migration, we used 3D collagen gels to mimic the interstitial microenvironment and cells were exposed to a diffusion gradient of CCL19. As shown in Figure 5 and Data S1, S2, S3, S4, and S5, mature DCs stimulated with LPS migrated efficiently toward the chemokine source. However, the chemotactic response of DCs infected with *O. tsutsugamushi* was significantly impaired and similar to that of immature DCs (*p* = 0.864). Interestingly, directional migration was slightly increased in DCs stimulated with UV-inactivated bacteria (*p*<0.001 vs. CNT, Figure 5A and B), suggesting that viability of the intracellular pathogen may affect its inhibition of DC migration. However, when we further stimulated *O. tsutsugamushi*-infected DCs with LPS, chemotatic migration recovered and was as efficient as LPS-stimulated DCs, suggesting that the impaired migration of infected DCs might not be due to an irreversible inhibition by the bacteria.

**Figure 6:**
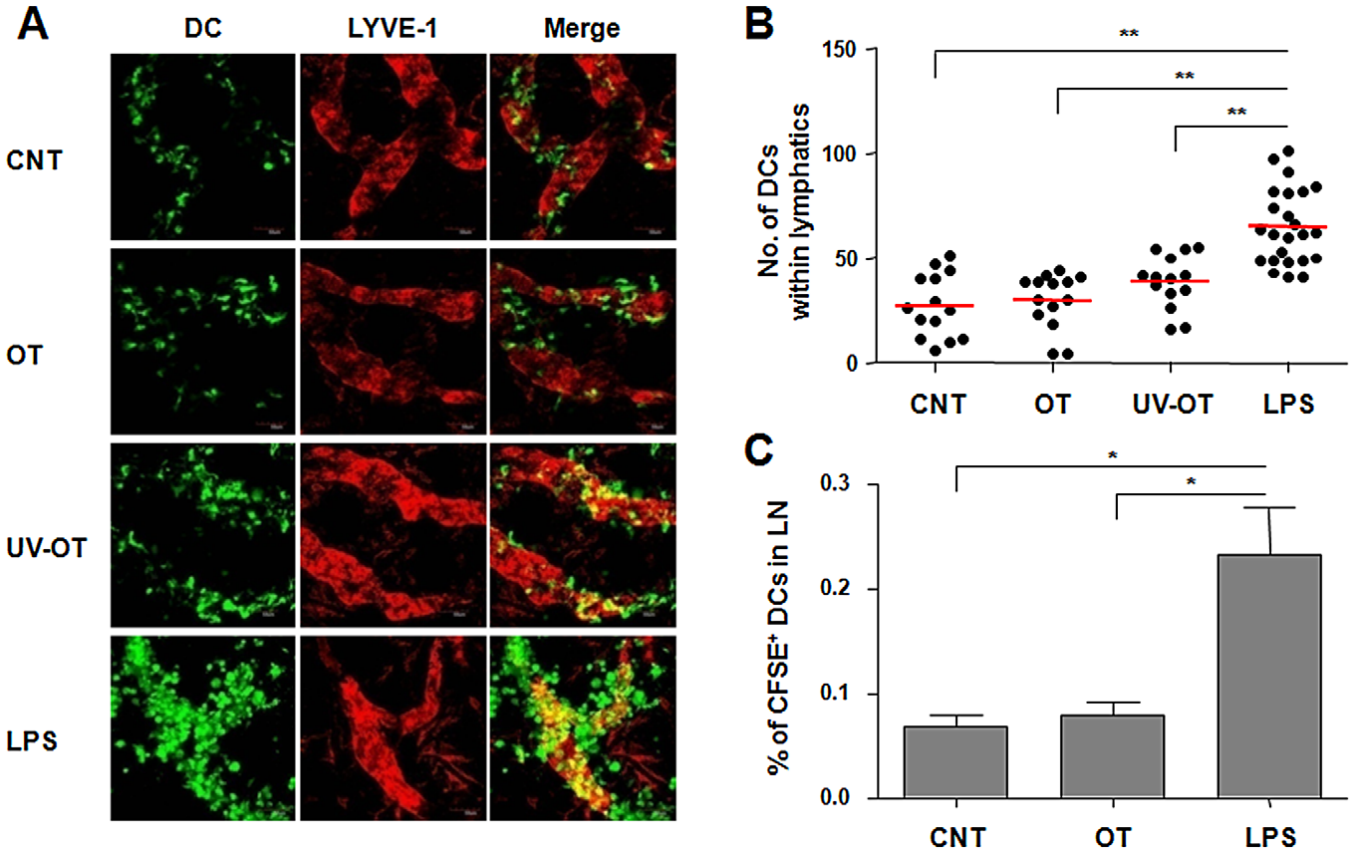
*Ex vivo* and *in vivo* migration of DCs infected with *O. tsutsugamushi*. A. Ears of C57BL/6 mice were split into halves and CFSE-labeled DCs (green) were put on top of the dermis. Colocalization of DCs within LYVE-1^+^ lymphatic vessels (red) in dermis was analyzed by confocal microscopy after 2 h of incubation. B. The numbers of DCs within lymphatics of ear dermis were counted in different fields of confocal images from A. **: *p*<0.01. Red bars represent mean values. C. *In vivo* migration of DCs into lymph nodes was analyzed after subcutaneous injection of CFSE-labeled DCs into hind footpads of C57BL/6 mice. At 2 days after injection, CFSE-labeled CD11c^+^ cells in draining popliteal lymph nodes were detected by flow cytometer. Mean percentile of CFSE-labeled DCs in total lymph node cells from three separate experiments are presented. Error bar: mean+S.D., *: *p*<0.05. CNT: immature DCs, OT: DCs infected with *O. tsutsugamushi*, UV-OT: DCs stimulated with UV-inactivated *O. tsutsugamushi*, LPS: DCs stimulated with LPS.

In order to examine the entry of DCs into lymphatic vessels, we used crawl-in assays in which fluorescently labeled DCs were placed on the dermis of ear explants. After incubation for 2 h, the numbers of DCs localized within the LYVE-1^+^ lymphatic vessels were counted and compared (Figure 6A and B). DCs stimulated with LPS were more efficiently localized within the lymphatic vessels than immature control DCs (*p*<0.001). In contrast, co-localization of *O. tsutsugamushi*-infected DCs was comparable to that of immature cells (*p* = 0.627). DCs stimulated with UV-inactivated bacteria showed a slight increase in the number of cells colocalized within lymphatic vessels, but this was statistically not significant (*p* = 0.068, Figure 6B). We further confirmed inefficient migration of DCs infected with *O. tsutsugamushi in vivo* (Figure 6C). Fluorescently labeled DCs were injected in the footpads of mice and their popliteal lymph nodes were analyzed at 2 days after injection. Approximately three times more DCs were detected in the draining lymph nodes when stimulated with LPS compared to unstimulated immature DCs, whereas migration of *O. tsutsugamushi*-infected DCs was similar to unstimulated immature DCs (*p* = 0.508).

### Comparison of CCR7 Surface Expression on DCs

**Figure 7:**
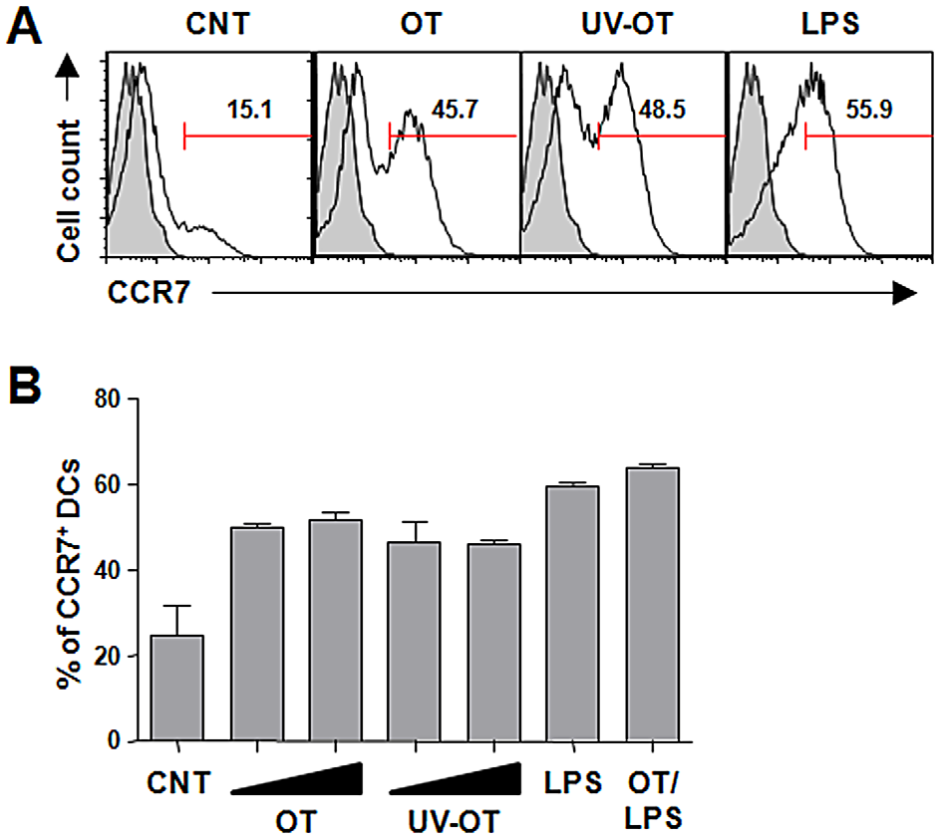
CCR7 expression on DCs infected with *O. tsutsugamushi*. A. Representative histograms of CCR7 surface expression on DCs at 20 h after the indicated stimulation are presented. The numbers within the graphs indicate the percentage of CCR7^+^ DCs. Gray histogram: isotype control. B. Percentage of CCR7^+^ DCs after stimulation was examined in three separate experiments. Increasing amounts (10 or 20 bacteria/cell) of bacteria were also used to detect dose-dependent responses. Error bar: mean+S.D., CNT: immature DCs, OT: DCs infected with *O. tsutsugamushi*, UV-OT: DCs infected with UV-inactivated *O. tsutsugamushi*, LPS: LPS-stimulated DCs.

Since the migration of DCs to regional lymph nodes requires the expression of CCR7, the receptor for lymphoid chemokines CCL19 and CCL21, we next analyzed the effect of *O. tsutsugamushi* infection on CCR7 surface expression in DCs. We found a portion (24.8±11.6%) of immature DCs with surface CCR7 expression in our experimental setup and mature DCs stimulated with LPS showed a remarkable increase in CCR7 surface expression (60.0±5.1%) (Figure 7). When DCs were infected with *O. tsutsugamushi*, CCR7-positive cells (52.2±2.8%) were significantly increased compared to that of immature control cells. Secondary stimulation of *O. tsutsugamushi*-infected DCs with LPS further increased the population of CCR7-positive cells (63.6±1.8%). Since we detected two populations of DCs in terms of CCR7 expression level in *O. tsutsugamushi*-infected DCs, we examined whether this is dose-dependent. The upregulation of CCR7 expression was not significantly changed when the cells were infected with increasing amounts of bacteria nor UV-inactivated ones (Figure 7B). Taken together, *O. tsutsugamushi* infection can induce surface expression of CCR7 in DCs and the impaired migration of DCs infected with *O. tsutsugmushi* might not be due to insufficient surface expression of CCR7.

### Differential Activation of MAP Kinases by CCL19 in DCs Infected with *O. tsutsugamushi*

**Figure 8:**
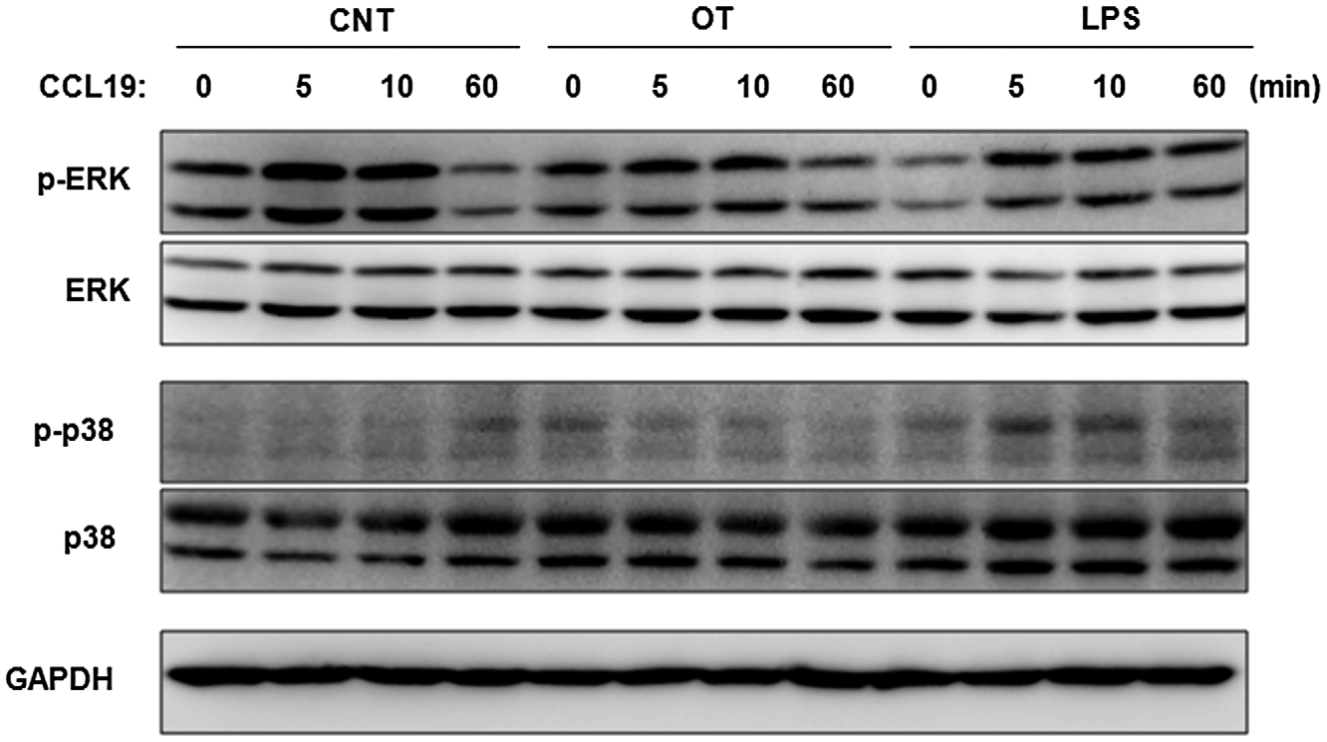
Differential activation of MAP kinases in *O. tsutsugamushi*-infected DCs upon exposure to CCL19. DCs were stimulated with *O. tsutsugamushi* (OT), or LPS for 18 h and then further incubated with CCL19 (200 ng/ml) for the indicated times. The activation of ERK and p38 MAP kinases was assessed by immunoblot using specific anti-phospho-ERK1/2 or phospho-p38 MAP kinases antibodies. ERK1/2, p38, and GAPDH were used as loading controls. CNT: immature DCs.

It was previously reported that engagement of CCR7 by its ligands, such as CCL19, activates MAP kinase members and this signaling pathway subsequently regulates chemotaxis of DCs [23]. Thus, we analyzed whether CCR7 induces activation of MAP kinases in *O. tsutsugamushi*-infected DCs upon exposure to CCL19. DCs stimulated with *O. tsutsugamushi* or LPS were incubated with CCL19 (200 ng/ml) for the indicated time periods. The cells were lysed, and analyzed by immunobloting using antibodies specific for the phosphorylated/active forms of MAP kinases, ERK and p38 (Figure 8). Treatment with CCL19 resulted in a rapid and potent activation of ERK in DCs. Phosphorylation of ERK reached a maximum after 5 to 10 min of incubation and returned to levels close to baseline by 60 min in immature control DCs and *O. tsutsugamushi*-infected DCs, but was sustained for 60 min in DCs stimulated with LPS. Interestingly, phosphorylation of p38 was barely detectable in control DCs and *O. tsutsugamushi*-infected DCs, in contrast to LPS-stimulated DCs which showed a transient activation of p38 after 5–10 min of incubation with CCL19. These results suggest that a differential activation of MAP kinase members upon chemokine exposure may contribute to the inefficient chemotaxis observed in *O. tsutsugamushi*-infected DCs.

## Discussion

Recent studies affirm the immunostimulatory activities of DCs such as increased expression of MHC complexes, upregulation of costimulatory molecules, and secretion of cytokines needed for efficient T cell priming [24]. Similar to cells infected with other *Rickettsia* species, activation markers were significantly upregulated after DCs were stimulated with *O. tsutsugamushi* (*p*<0.05) [25]. However, the level of DC activation, as measured by the surface expression of MHC II and costimulatory molecules, was significantly lower than those of cells stimulated with LPS (*p*<0.05). Previously, it was shown that *O. tsutsugamushi* lacks LPS and peptidoglycan in its cell wall [26], [27]. In addition, the genes for the synthesis of such cell components are absent in the bacterial genome [8], [27]. Thus, the absence of cell wall components, especially LPS, makes *O. tsutsugamushi* less stimulating to DCs and may explain the “semi-mature” phenotypes of DCs after bacterial infection. It could be postulated that the bacteria evolved to modify their envelope structures to be specific to the host to which they have adapted [8], [27], [28].

The secretion of chemokines and cytokines by activated DCs is critical in orchestrating T cell responses in regional lymph nodes. Previously, it was reported that *O. tsutsugamushi* induces various chemokines in macrophages and endothelial cells via the activation of NF-κB and AP-1 transcription factors [13], [29], [30], [31]. In a macrophage cell line, mRNAs encoding MIP-1α/β, MIP-2, and MCP-1 were increased in response to *O. tsutsugamushi* infection [13]. In addition, the expression of chemokines Ltn, RANTES, MIP-1α, MIP-1β, MIP-2, and MCP-1 and cytokines LTβ, TNF-α, IL-6, IFN-γ, TGF-β1, and MIF was upregulated in mice infected with *O. tsutsugamushi* [32]. Here, we also observed increased secretion of IL-6, IL-12, MIP-1α, and RANTES in *O. tsutsugamushi*-infected DCs. These results suggest that even though *O. tsutsugamushi* lacks LPS, it is capable of activating DCs to a certain extent, inducing inflammatory responses potentially via the activation of NF-κB and AP-1 transcription factors. Given that the migration of DCs infected with *O. tsutsugamushi* was considerably impaired, preferential secretion of the CC chemokine subfamily, which includes RANTES and MIP-1α from *O. tsutsugamushi*-infected DCs at the infection site may contribute to the infiltration of monocytes and lymphocytes into the infection sites as observed in the eschars from scrub typhus patients [9].

Autophagy is recognized to be a *bona fide* immunological process with a wide array of roles in immunity against intracellular pathogens [33]. It is a specialized cytoplasmic system for the direct elimination of intracellular bacteria and a crucial contributor to intracellular antigen processing and MHC presentation of endogenously expressed antigens [34], [35], [36], [37]. Recently, it was also shown that cellular autophagy is essential for innate cytokine production and APC functions in DCs infected with pathogenic viruses [38], [39], [40]. Therefore, autophagosomes induced by pathogenic infection play a pivotal role in both innate and adaptive host defenses against intracellular pathogens. Here, we show for the first time that live *O. tsutsugamushi* actively evades the cellular autophagy system, although it induces autophagosomes in DCs regardless of its viability (Figure 4). This active escape from induced autophagy may significantly affect the APC functions of DCs, which not only includes its ability to directly kill the intracellular pathogen via autophagosomal degradation, but also antigen processing and presentation, both essential for the subsequent induction of adaptive immunity in the infected host. Various intracellular bacteria have developed sophisticated mechanisms to escape from the autophagic machinery. *Shigella* IscB protein mediates active escape from autophagy by competitively inhibiting the interaction of VirG bacterial protein with host Atg5, which is required for autophagy induction [41]. *Listeria* ActA and InlK proteins promote the recruitment of host cytosolic proteins to the bacterial surface in order to mask the pathogen from recognition by the autophagic system, thereby promoting intracellular survival [42], [43]. Given that most live *O. tsutsugamushi* actively escape induced autophagosomes within the cytosol of DCs, the intracellular pathogen may also be equipped with an efficient mechanism of autophagy evasion that remains to be elucidated.

Antigen-presenting DCs acquire foreign antigens in peripheral tissues. Efficient DC migration to draining lymph nodes through lymphatic vessels optimizes foreign antigen presentation to naive T cell [44]. DC migration into and along this conduit occurs through a series of steps including mobilization, detachment, interstitial migration, entry into the afferent lymphatics, and transit via lymph [24]. Within an artificial 3D matrix of collagen, DCs stimulated with LPS migrated along gradients of CCL19 and showed amoeboid morphology and velocities that were comparable to *in vivo* observations (Figure 5 and Data S1, S2, S3, S4, and S5) [17]. However, the chemotactic response of DCs infected with *O. tsutsugamushi* was drastically impaired and similar to that of un-stimulated immature DCs. Moreover, the impaired migration of *O. tsutsugamushi* infected-DCs was consistent in both *ex vivo* and *in vivo* experimental settings (Figure 6). These results suggest that *O. tsutsugamushi* may actively inhibit the chemotactic migration of DCs or does not stimulate the cells as efficiently as LPS, a strong DC activator. To test this hypothesis, we stimulated *O. tsutsugamushi*-infected DCs with LPS. This secondary stimulation restored efficient chemotatic migration of DCs infected with *O. tsutsugamushi* (Figure 5) as well as further upregulation of costimulatory molecules on DCs (Figure 1). The restoration of chemotatic migration by the secondary LPS stimulation was also observed even after extended incubation (up to 8 h) with *O. tsutsugamushi* (Data S6). Therefore, *O. tsutsugamushi* suboptimally stimulates DCs rather than irreversibly inhibits DC activation and migration.

DC migration is primarily guided by the two chemokines, CCL19 and CCL21, which are expressed in lymphatic endothelium and the T cell area of lymph nodes [17]. Antigen uptake by DC induces maturational changes that include decreased expression of the chemokine receptors CCR1, CCR2, and CCR5 that maintain DC residence in peripheral tissues, and increased expression of CCR7 that mediates the migration of antigen-bearing DC to lymphatic tissue via binding to the chemokines [6]. When we examined the surface expression of CCR7 on DCs infected with *O. tsutsugamushi*, the level of CCR7 expression was comparable to that of LPS-stimulated DCs (Figure 7), suggesting that suboptimal expression of CCR7 on DCs is not the cause of impaired chemotactic migration of *O. tsutsugamushi*-infected DCs. Various microbial pathogens have established diverse strategies to control DC migration. Human cytomegalovirus (HCMV) blocks the migration of infected monocyte-derived DCs toward lymphoid chemokines, CCL19 and CCL21, by modulating the level of CCR7 expression [45]. HCMV very efficiently triggers the down regulation of CCR5 without inducing the expression of CCR7 in infected DCs, even following stimulation with LPS/TNF-α/IFN-γ, normally a potent stimulus for CCR7 induction [45]. In the case of human metapneumovirus (HMPV) and human respiratory syncytial virus (HRSV), viral infection of monocyte-derived DCs did not efficiently decrease CCR1, 2, and 5 expression, and did not efficiently increase CCR7 expression [46]. The inefficient chemokine receptor modulation of DCs by viral infection results in poor migration toward the CCR7 ligand, CCL19. HMPV- or HRSV-stimulated DCs responded to secondary stimulation with LPS or a cocktail of proinflammatory cytokines by increasing CCR7 and decreasing CCR1, 2 and 5 expression, and by more efficient migration to CCL19, suggesting that HMPV and HRSV suboptimally stimulate rather than irreversibly inhibit DC migration. When we analyzed the surface expression of chemokine receptors, CCR5 was efficiently downregulated (data not shown) and CCR7 was upregulated in *O. tsutsugamushi*-infected DCs, suggesting that decreased cheomotactic migration is not due to impaired chemokine receptor modulation in *O. tsutsugamushi*-infected DCs.

Several studies have proposed that integrated signaling modules regulate chemotaxis in CCR7-stimulated DCs [23], [47]. Various non-overlapping signaling modules, including Gαi-mediated activation of p38 and ERK1/2, appear to regulate chemotactic migration of DCs induced by CCR7 chemokine ligands [47]. Recently, expression of CCR7 alone was shown to be insufficient for DC migration, as it can be expressed in a biologically insensitive state such that CCR7^+^ DCs either fail to undergo chemotaxis towards CCR7 ligands [48] or require a high concentration of CCR7 ligands before they respond [44]. The mechanisms by which these mediators alter CCR7 functionality is not known, but they probably trigger signaling events that, in turn, alter the signaling cascades that are engaged when CCR7 binds to its ligands [49], [50]. In this study, phosphorylation of p38 was barely detectable in *O. tsutsugamushi*-infected DCs as well as in control DCs, in contrast to the LPS-stimulated DCs which showed a transient activation of p38 after 5–10 min of incubation with CCL19. Given that phosphorylation of ERK occurs in both uninfected and infected DCs (Figure 8), the specific failure of p38 activation in *O. tsutsugamushi*-infected DCs may be the result of the “semi-mature” phenotypes and impaired migration of the infected DCs. Interestingly, inhibition of all these MAP kinases does not completely blunt CCR7-dependent chemotaxis [23], [51], suggesting that additional unidentified molecules or signaling pathways may also regulate chemotaxis of DCs [52]. In order to elucidate the precise molecular mechanisms that control DC migration during *O. tsutsugamushi* infection, further studies are needed.

Recently, a paper reported that *O. tsutsugamushi* mainly infects “inflammatory” CD14/LSP-1/CD68 positive monocytes and CD1a/DCSIGN/S100/FXIIIa and CD163 positive dendritic cells in stained eschar skin biopsies from scrub typhus patients [9]. Not only that, innate APCs were highly accumulated in these pathologic lesions. The authors propose that infection of dendritic cells and activated inflammatory monocytes offers a potential route for dissemination of *O. tsutsugamushi* from the initial eschar site. The immunomodulatory effects of *O. tsutsugamushi* infection on local APCs in the eschar site could also interfere with downstream host immune responses. Our current results further suggest that *O. tsutsugamushi* infection might interfere with the active exit of DCs from the initial infection site and exploit these sentinel cells as a reservoir for bacterial replication. The functional exploitation of DCs by *O. tsutsugamushi* may contribute to bacterial pathogenesis during the early phase of infection and interfere with effective downstream adaptive immunity required for protection against bacterial infection. Further studies on the effect of *O. tsutsugamushi* infection on adaptive immune responses, especially on antigen-specific T cell immunity in conjunction with impaired DC functions by bacterial infection, may enhance our understanding of immunological pathogenesis in scrub typhus patients.

## Supporting Information

Data S1 **In vitro migration of unstiumlated immature DCs toward CCL19 gradient in a 3D collagen matrix was monitored for 4 h.**
(MOV)

Data S2 **In vitro migration of DCs stimulated with LPS toward CCL19 gradient in a 3D collagen matrix was monitored for 4 h.**
(MOV)

Data S3 **In vitro migration of DCs infected with live O. tsutsugamushi toward CCL19 gradient in a 3D collagen matrix was monitored for 4 h.**
(MOV)

Data S4 **In vitro migration of DCs stimulated with UV-inactivated O. tsutsugamushi toward CCL19 gradient in a 3D collagen matrix was monitored for 4 h.**
(MOV)

Data S5 **In vitro migration of DCs stimulated with live O. tsutsugamushi and LPS toward CCL19 gradient in a 3D collagen matrix was monitored for 4 h.**
(MOV)

Data S6 ***In vitro*** **chemotactic migration of DCs incubated with** ***O. tsutsugamushi*** **for indicated time period and subsequently stimulated with LPS for 20 h was monitored in a 3D collagen matrix.** Single cell tracking was performed using Manual Tracking Plugin with Image J software. Thirty cells were randomly selected and tracked for 4 h.
(PPT)
