# Supplementary figures and images for: Orientia tsutsugamushi Subverts Dendritic Cell Functions by Escaping from Autophagy and Impairing Their Migration
Source: PLoS Negl Trop Dis. 2013 Jan 3;7(1):e1981. doi: 10.1371/journal.pntd.0001981 (PMC3536799; doi:10.1371/journal.pntd.0001981)

## Slide 1
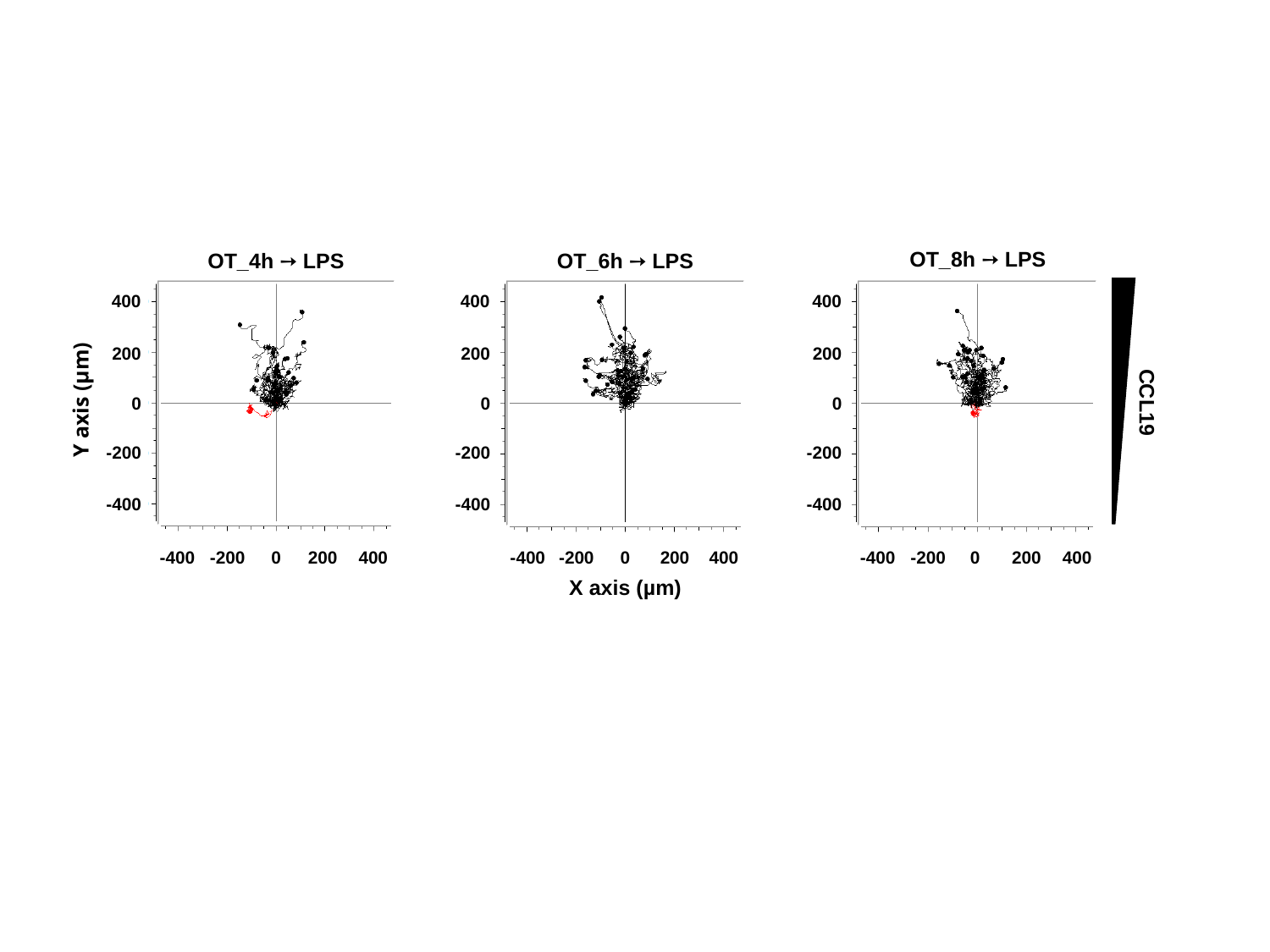

OT_8h ➙ LPS
OT_4h ➙ LPS
OT_6h ➙ LPS
400
400
400
200
200
200
Y axis (µm)
0
0
0
-200
-200
-200
-400
-400
-400
X axis (µm)
-400
-200
0
200
400
-400
-200
0
200
400
-400
-200
0
200
400
CCL19

Supplement: Data S6 — In vitro chemotactic migration of DCs incubated with O. tsutsugamushi for indicated time period and subsequently stimulated with LPS for 20 h was monitored in a 3D collagen matrix. Single cell tracking was performed using Manual Tracking Plugin with Image J software. Thirty cells were randomly selected and tracked for 4 h. (PPT) [file pntd.0001981.s006.ppt]
